# C1-inhibitor/C1-inhibitor antibody complexes in acquired angioedema due to C1-inhibitor deficiency

**DOI:** 10.1186/s13023-023-02625-5

**Published:** 2023-02-01

**Authors:** Zsofia Polai, Erika Kajdacsi, Laszlo Cervenak, Zsuzsanna Balla, Szabolcs Benedek, Lilian Varga, Henriette Farkas

**Affiliations:** 1 Department of Internal Medicine and Haematology, Hungarian Angioedema Center of Reference and Excellence, Semmelweis University, Szentkiralyi u. 46, Budapest, 1088 Hungary; 2 Department of Internal Medicine and Haematology, Semmelweis University, Budapest, Hungary

**Keywords:** Bradykinin-mediated angioedema, Acquired angioedema due to C1-inhibitor deficiency, C1-inhibitor, Antibodies against C1-inhibitor, Complement cascade, Classical pathway, Lymphoproliferative diseases

## Abstract

**Background:**

Autoantibodies against C1-inhibitor (C1-INH-Ab) have a diagnostic value in acquired angioedema due to C1-inhibitor deficiency (C1-INH-AAE), even though antibodies can circulate in complexes, which can be undetectable by proven methods. Our aim was to measure C1-INH/C1-INH-Ab complexes (CAC) and investigate their connection to C1-INH-Ab and the changes in their titer over time.

**Results:**

19 patients were diagnosed with C1-INH-AAE in the Hungarian Angioedema Center of Reference and Excellence; 79% of them had an underlying disease. Samples were examined with a newly developed in-house complex ELISA method. Patients with high C1-INH-Ab titer had a CAC titer which did not exceed the normal level and the ones with high CAC titer had a C1-INH-Ab titer which did not exceed the normal level. In case of those patients who had C1-INH-Ab and CAC of the same type of immunoglobulin, the increasing titer of C1-INH-Ab went together with the decreasing level of CAC and vice versa. CAC titer was already increased before the diagnosis of the underlying disease.

**Conclusions:**

Free circulating and complex antibodies are in a dynamically changing equilibrium. CAC measurements can help to predict the development of an underlying disease. The efficiency of the treatment for underlying disease can be monitored by the decreasing CAC titers.

Our results show that the CAC can be of important additional information besides the complement panel examination in case of C1-INH-AAE. Measurement of CAC is recommended to be done parallelly with C1-INH-Ab, so as to detect both free and bound antibodies.

## Background

Angioedema with C1-inhibitor (C1-INH) deficiency can be hereditary (C1-INH-HAE)—an autosomal dominant disorder caused by mutations in the *SERPING1* gene—or acquired (C1-INH-AAE). Both are rare diseases: the incidence of C1-INH-HAE is estimated at 1:10,000 to 1:50,000, whereas the prevalence of C1-INH-AAE is ten times lower [1].

C1-INH is a serpin-type protease inhibitor, which controls the classical and lectin complement pathways, the coagulation cascade, the fibrinolytic and kinin-kallikrein system [2–4]. C1-INH is one of the most abundant circulating protease inhibitors (0.25 g/L) [5], and its multifaceted significance is undeniable. It is the sole inhibitor of C1r and C1s and the major inhibitor of MASP-1 and MASP-2 in the complement system [6–8]. In the contact-kinin-kallikrein cascade system, it is the major inhibitor of plasma kallikrein [9] and factor XIIa [10]. It is the major inhibitor of coagulation factor XIa as well [11], and also inhibits thrombin [12].

C1-INH-AAE patients usually have a negative family history and in most patients their disease appears over the age of 40 years in the form of recurrent angioedematous episodes, involving the subcutanous and/or submucosal tissues [13, 14]. Laryngeal angioedema can be fatal if the patient does not receive adequate care in a timely manner [15]. Angioedema attacks can be triggered by mechanical trauma, emotional stress, and medications (especially angiotensin converting enzyme inhibitors) [16, 17].

Decreased concentration of antigenic C1-INH in C1-INH-AAE can be attributed to the increased utilization due to increased activation of the classical complement pathway or due to the production of autoantibodies against C1-INH (C1-INH-Ab) [18]. In the early phase of comprehending C1-INH-AAE, the disease was divided into two types: one based on the association of some (typically hematological) underlying disease and the other type defined by the presence of autoantibodies as an immune disease [13, 14, 19–24]. Subsequent research demonstrates an overlap between the two types: patients with detectable C1-INH-Ab may also develop underlying disease(s) [25–27].

C1-INH-AAE can be associated with several further diseases, mainly lymphoproliferative ones, like monoclonal gammopathy of undetermined significance (MGUS) and non-Hodgkin lymphomas [3, 22, 28, 29].

The diagnosis of C1-INH-AAE is based on the measurement of complement parameters: typically, the total hemolytic complement (CH50), C1-INH functional activity, C4, C1q, C1-INH antigenic concentration are decreased, C3 titer is either in the normal range or decreased. The presence of C1-INH-Ab has a diagnostic value [30–32]. The decrease of C1-INH antigenic concentration may be due to the neoplastic lymphatic tissue enhancing activation of the classical pathway or due to the presence of neutralizing C1-INH-Ab. C1q consumption is attributed to the uncontrolled activation of the complement classical pathway due to high titers of circulating C1-INH/C1-INH antibody complexes (CAC), eventually causing the exhaustion of C1-INH inhibitory activities [3, 30, 33]. C1-INH-Ab can interfere with the function of CI-INH, and C1-INH-Ab can recognize different epitopes within C1-INH [30, 33].

In case of C1-INH-AAE patients, free C1-INH is circulating in a cleaved/inactivated form [3, 26].

According to the theory of the idiotype-antiidiotype network, the antibodies produced by our immune system can connect not only to the antigen dedicated by the variable region of the antibody, but variable regions can connect to each other as well. Natural antibodies can occur in a cross-reaction with low affinity, while pathological antibodies usually have higher affinity and serum concentration. This is most common for IgM-type antibodies [34]. The formed immune complexes are able to activate the classical pathway of the complement system which leads to the elevated consumption of the proteins of the complement system.

Antibodies can be found not only in free circulating form, but also in complexes which means that measuring only the free form can cause an error in underestimating the actual amount of antibodies [34].

Our study aimed to use a new method for the measurement of CAC and aimed to uncover a possible connection between C1-INH-Ab and CAC and a pattern of CAC during long term follow-up connected to C1-INH-AAE and the related underlying disease.

## Results

The CAC measurements were performed by our newly developed ELISA method on 20 separate pooled normal serum samples (healthy control), their summarized result was taken as normal level.

In case of 11/19 patients C1-INH-Ab could be detected at least once during the follow-up period (Table 1).

**Table 1:** Antibodies against C1-inhibitor in our patient group

Antibodies against C1-inhibitor			
Patient ID	IgG	IgM	IgA
P1	–	–	–
P2	–	–	–
P3	–	–	–
P4	–	–	X
P5	–	X	–
P6	–	–	–
P7	–	X	–
P8	–	X	–
P9	–	–	X
P10	X	X	X
P11	X	X	–
P12	X	X	–
P13	–	–	–
P14	–	–	–
P15	X	–	X
P16	X	–	–
P17	–	–	X
P18	–	–	–
P19	–	–	–

–: antibody was not detected, X: antibody was detected at least once during the follow-up

5/19 patients had regularly (at least in 50% of the measuring occasions) C1-INH-Ab (Table 2).

**Table 2:** Free circulating and complex C1-inhibitor antibodies

	IgG C1-INH-Ab	CAC IgG	IgM C1-INH-Ab	CAC IgM	IgA C1-INH-Ab	CAC IgA
Free circulating and complex antibodies against C1-inhibitor						
P1	–	–	–	+	–	+++
P2	–	+	–	+++	–	+++
P3	–	+	–	++	–	+++
P4	–	–	–	–	–	–
P5	–	–	–	–	–	–
P6	–	–	–	+++	–	–
P7	–	++	–	+++	–	++
P8	–	–	–	+	–	+
P10	–	+++	++	++	–	+++
P11	+++	–	–	–	–	+++
P12	+++	–	++	–	–	–
P13	–	–	–	++	–	++
P14	–	–	–	+++	–	–
P15	+++	–	–	+++	–	–
P16	++	++	–	++	–	–
P17	–	–	–	+++	+++	++
P18	–	–	–	–	–	–
P19	–	–	–	+	–	–

*C1-INH-Ab* antibodies against C1-inhibitor, *CAC* C1-inhibitor/C1-inhibitor antibody complex, –: does not exceed the normal value with more than 20%, + : exceeds the normal value with 20–40%, ++ : exceeds the normal value with 40–60%, +++ : exceeds the normal value with more than 60%

11/19 patients had free C1-INH-Ab, 14 patients CAC, 3 patients neither C1-INH-Ab nor CAC, and 5 patients C1-INH-Ab as well as CAC. One patient had only C1-INH-Ab and no CAC, and 8 patients only CAC and no C1-INH-Ab.

We gave one word description for the C1-INH-Ab and CAC results based on what was characteristic for the patient’s value ’in the long run (which generally describes the results during the follow-up) (Table 3). ‘–’ means that the results exceeded the normal level with maximum 20%; ‘+’ means that the results are 20–40% above the normal level;‘++’ means 40–60% higher results than normal level and ‘ +++ ’ means more than 60% more than the normal level.

**Table 3:** Disease background of our patients

Patient ID	Gender	Age at the first angioedema attack	Underlying disorders	Treatment of underlying disorders
P1	M	60	–	–
P2	F	40	Marginal zone lymphoma	–
P3	F	83	MGUS	–
P4	F	–	–	–
P5	F	–	Non-Hodgkin lymphoma	Rituximab, ASCT
P6	F	56	Marginal zone lymphoma	Rituximab
P7	F	61	Chronic lymphoid leukemia	Rituximab
P8	M	49	MGUS	–
P9	F	65	Basal cell adenoma	–
P10	M	70	MGUS	Rituximab
P11	F	49	MGUS	–
P12	M	59	Myeloma multiplex	Chemotherapy (VAD), ASCT, rituximab
P13	M	46	Marginal zone lymphoma	Rituximab
P14	F	71	Marginal zone lymphoma	Rituximab
P15	M	60	–	–
P16	M	42	MGUS	–
P17	M	76	MGUS	–
P18	F	71	Marginal zone lymphoma	Rituximab
P19	M	43	–	–

*M* male, *F* female, *AAE* acquired angioedema, *MGUS* monoclonal gammopathy of undetermined significance, *ASCT* autologous stem cell transplantation, *VAD* vincristine, doxorubicin, dexamethasone

**Fig. 1:**
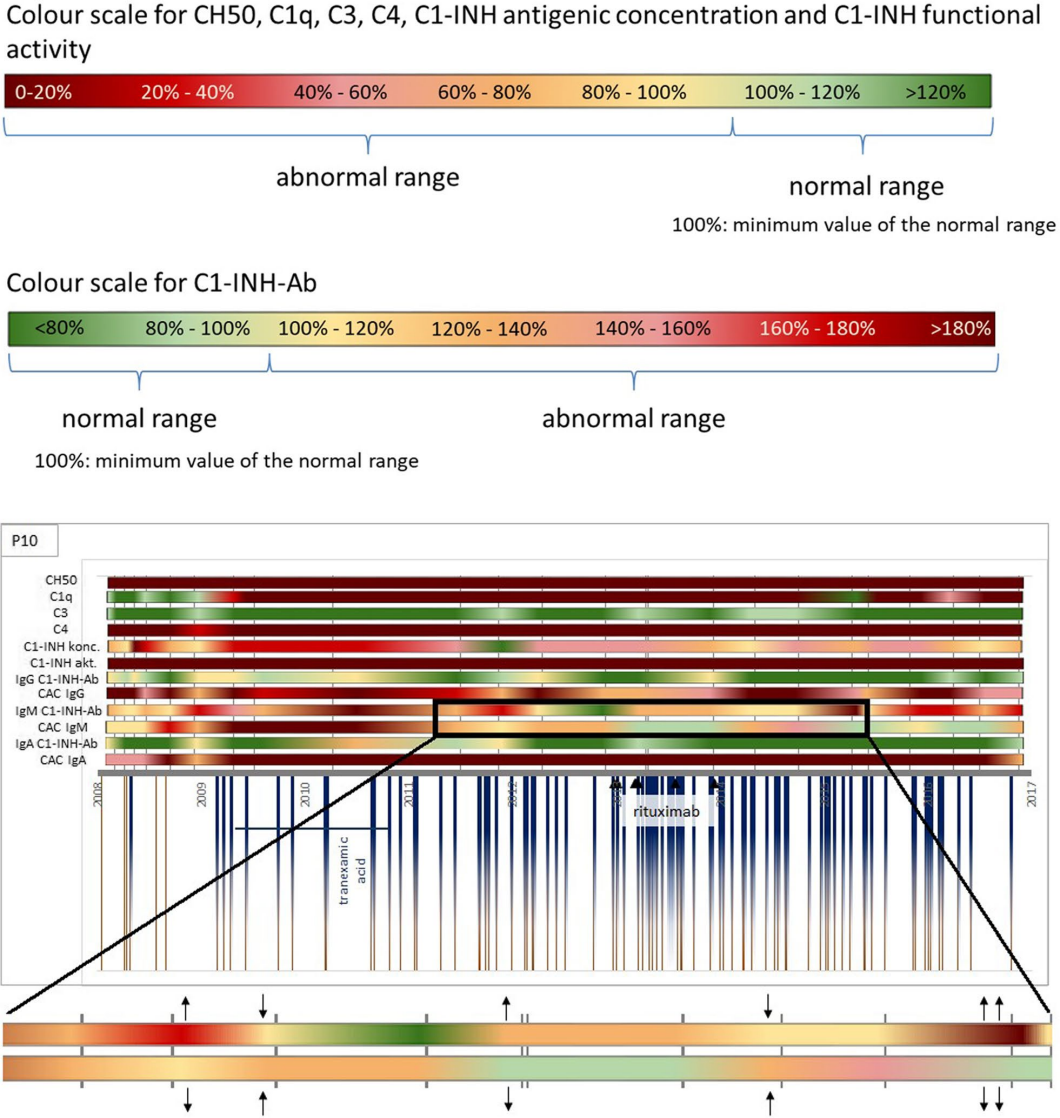

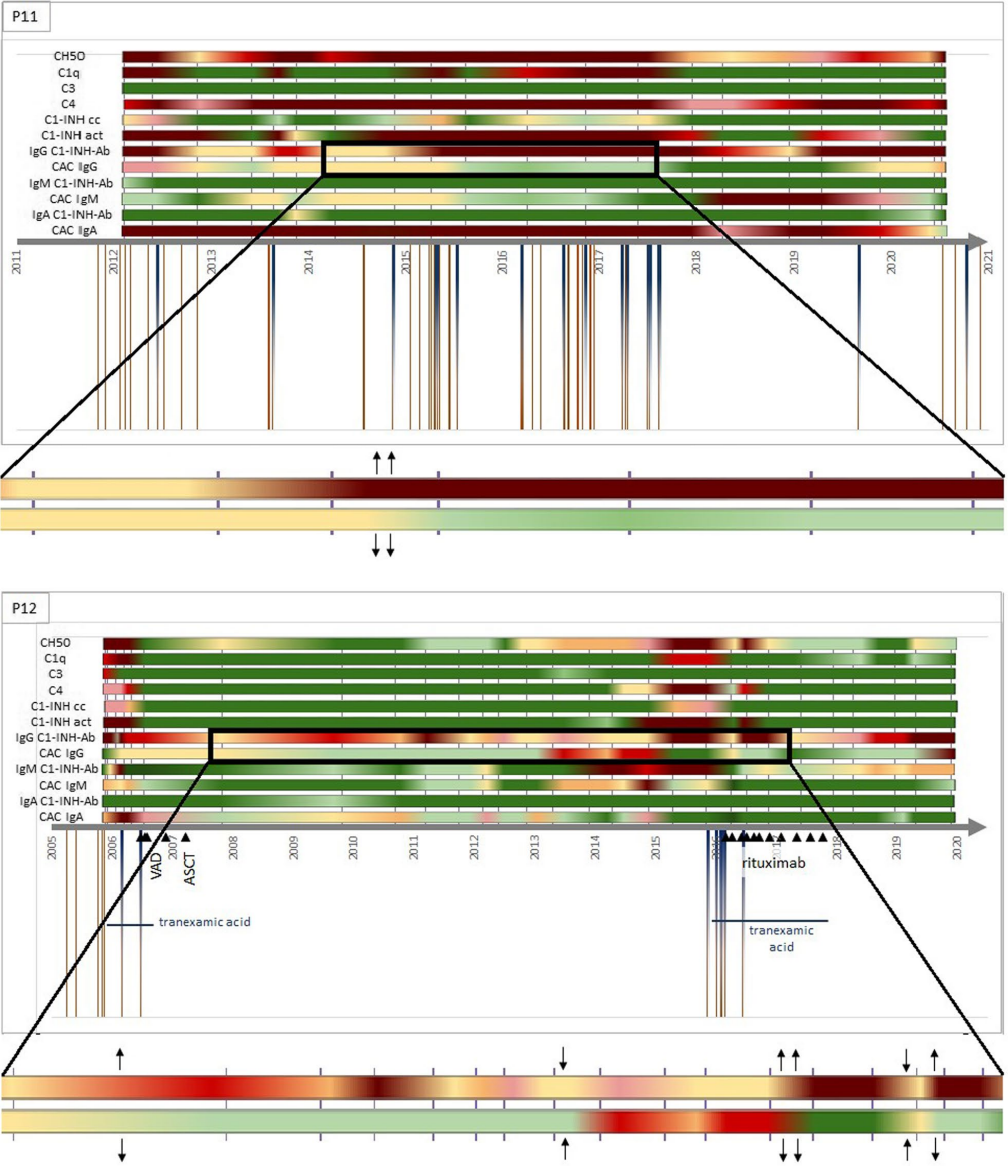
Changes of complement parameters, angioedema attacks and treatments during long term follow-up. CH50: total hemolytic complement, C1-INH cc: antigenic C1-inhibitor concentration, C1-INH act: C1-inhibitor activity, C1-INH-Ab: antibody against C1-inhibitor, CAC: C1-inhibitor/C1-inhibitor antibody complex, ↑: increasing titer of C1-INH-Ab or CAC, ↓: decreasing titer of C1-INH-Ab or CAC, VAD: chemotherapy of vincristine, doxorubicin, dexamethasone; ASCT: autologous stem cell therapy

For a more detailed examination and illustration we chose 3 patients, who had at least 8 years of follow-up and had elevated C1-INH-Ab and CAC titers. (Fig. 1) Over the time axis, the change of certain complement parameters can be seen during the multiannual follow-up period. The vertical grey lines (behind the colourful lines) mark the sampling points. On the top of the figure, a colour code can be seen: green colors mean that the given parameter was in the normal range; from yellow to red, the values are further away from the normal range. Under the time axis, brown lines mean the attacks, and blue half-lines mean the treatments. Horizontal blue lines show a long-term prophylaxis, with the medication named under. Black triangles show the time of the underlying disease treatment. The deceased patient’s timelines end without an arrow.

A closer look on the timelines leads us to the observation that in patients with both C1-INH-Ab and CAC forms of a given immunoglobulin type, these parameters change together: as one decreases, the other increases and vice versa. For easier understanding, a part of the timeline is enlarged and the increase (↑) and decrease (↓) of the titer are marked by arrows. This shows that free circulating and complex antibodies create a dynamically changing equilibrium state and that the absolute amount of antibodies can be approximately constant.

Two of the 19 patients’ (P10, P12) results show that the CAC titer is already increased before the diagnoses of the underlying disease.

## Discussion

C1-INH-AAE is a rare disorder and relevant information on it is scarce owing to the limited number of patients. In our Center, the ratio of patients with C1-INH-AAE and with C1-INH-HAE is 1 to 11 within the Hungarian patient population. This corresponds to data in the literature [14, 26, 35]. While in case of C1-INH-HAE the follow-up of the complement parameters is not necessary, in C1-INH-AAE, this follow-up gives remarkable help for the physicians to determine and predict the state of the disease.

The appearance of MGUS is already a reason for a detailed hematological examination [14, 36]. Among our patients, the incidence of underlying disease was 79%, in the literature the data ranges from 79 to 90% [14, 37].

In case of 11/19 patients C1-INH-Ab was detected at least once during the follow-up period. In percentage this means 58%, while Bork reported 44% as the incidence of C1-INH-Ab [14]. 5/19 patients (26%) regularly had C1-INH-Ab.

A study targeting CAC has so far occurred in only one Spanish publication in the literature [3].

Similarly to this study, there were patients in our patient group who had C1-INH-Ab and no CAC, some of them did not have C1-INH-Ab but had CAC, some of them had both or neither form of antibodies. An interesting observation is that those who had a high titer of C1-INH-Ab had a titer of CAC that did not exceed the normal level, and those who had a high titer of CAC, their titer of C1-INH-Ab did not exceed the normal level. Our observations show that free circulating and complex antibodies create a dynamically changing equilibrium state and that the absolute amount of antibodies can be approximately constant. The two patients (P4, P5) with C1-INH deficiency who did not experience angioedema did not have elevated C1-INH-Ab or CAC titers.

Results show that the CAC titer is already increased before the diagnosis of the underlying disease. CAC measurements can help to predict the development of an underlying disease and the efficiency of the treatment for underlying disease can be monitored by the decreasing CAC titer (P12 after rituximab).

In general, complex formation can activate the classical pathway thus serving as a positive feedback and also contributing to the elevated consumption of the elements of the pathway.

## Conclusions

Changes in CAC titers are suitable for monitoring C1-INH-AAE and the connecting underlying disease.

Examination of CAC titer has an importance beside testing C1-INH-Ab, because patients with elevated CAC titer, might have normal C1-INH-Ab titer. Measurement of CAC complexes is recommended parallelly with C1-INH-Ab, so as to detect both free and bound forms of antibodies. Free circulating and antibodies in complex create a dynamically changing equilibrium.

With the long-term follow-up of complexes, underlying diseases can be monitored from a new aspect, and the efficiency of their treatments could be determined.

CAC testing can give important additional information beside complement panel examination in case of C1-INH-AAE patients.

Our study offers a more efficient treatment for C1-INH-AAE patients, a more accurate, more reliable, and objective follow-up of the disease. With the help of our method, the course of the disease becomes more predictable, which provides safety to the treating physician and the patient, and the early diagnosis of the underlying disease increases the chance of survival, improves the quality of life and decreases the load on the healthcare system.

## Methods

### Patients

Due to the rarity of C1-INH-AAE, we have diagnosed, treated, and monitored 19 patients with this disease in the Hungarian Angioedema Center of Reference and Excellence in the past 30 years. There was no justification to exclude any patient from this study.

All patients were real time monitored. Follow-up visits took place at least once in 6 months in the first two years after the diagnosis of C1-INH-AAE, and at least once a year afterwards.

79% of the patients had an underlying disease, which are listed in Table 3. Treatments for the underlying disease (if there was any) can be found in Table 3 as well. Two patients (P4, P5) did not experience angioedema attacks neither before the diagnosis of acquired C1-INH deficiency (complement measurement was performed the first time due to their related disease), nor during the follow-up period.

Patient samples were selected from the last 15 years (2005–2020) and they were stored at − 80 °C until usage.

Healthy control samples (pooled normal serum) were stored on − 80 °C for less than 1 month.

### Complement measurements

The following complement parameters were measured right after sampling. To quantify C1-INH concentration, in-house radial immunodiffusion was performed. The level of C1-inhibitor activity was measured with a C1-inhibitor enzyme immunoassay kit (Quidel, San Diego, CA). The concentration of C1q and autoantibodies against C1-INH were determined by in-house sandwich ELISA methods. [38, 39] CH50 (total hemolytic activity) of the classical pathway was determined with a hemolytic assay. C3 and C4 concentrations were measured by turbidimetry (Cobas Integra 400 analyzer; Roche, Switzerland).

### CAC measurements

CAC measurements were performed on samples, which were not thawed before.

The measurement was performed on a 96-well Nunc Maxisorp (ThermoFisher, USA) plate, coated by rabbit anti-human C1-inhibitor IgG antibody purified by affinity chromatography. Coating was performed on 37 °C for 2 h or on 4 °C overnight. After coating and threefold washing, the plate was blocked by 2% BSA-PBS (bovine serum albumin—phosphate buffered saline) for 2 h.

After a threefold washing step, the blank, the controls and the samples were placed on the plate for 1 h. 1% BSA-PBS-Tween was used as blank and for diluting the samples and controls. Samples and the normal serum control were measured in 1000-fold dilution. After a threefold washing step, the conjugates were horseradish peroxidase labeled rabbit anti-human antibodies against IgG, IgM and IgA antibodies, which were incubated on the plate for 1 h on room temperature. Threefold washing step was followed by TMB (3,3′5,5′-Tetramethylbenzidine) detection, and after a few minutes of incubation on room temperature the stop solution (0,4 M sulfuric acid) was added. Absorbance was measured on 450 nm, reference measurement was performed on 620 nm by Tecan device (Tecan M1000pro, Group Ltd).

**Fig. 2:**
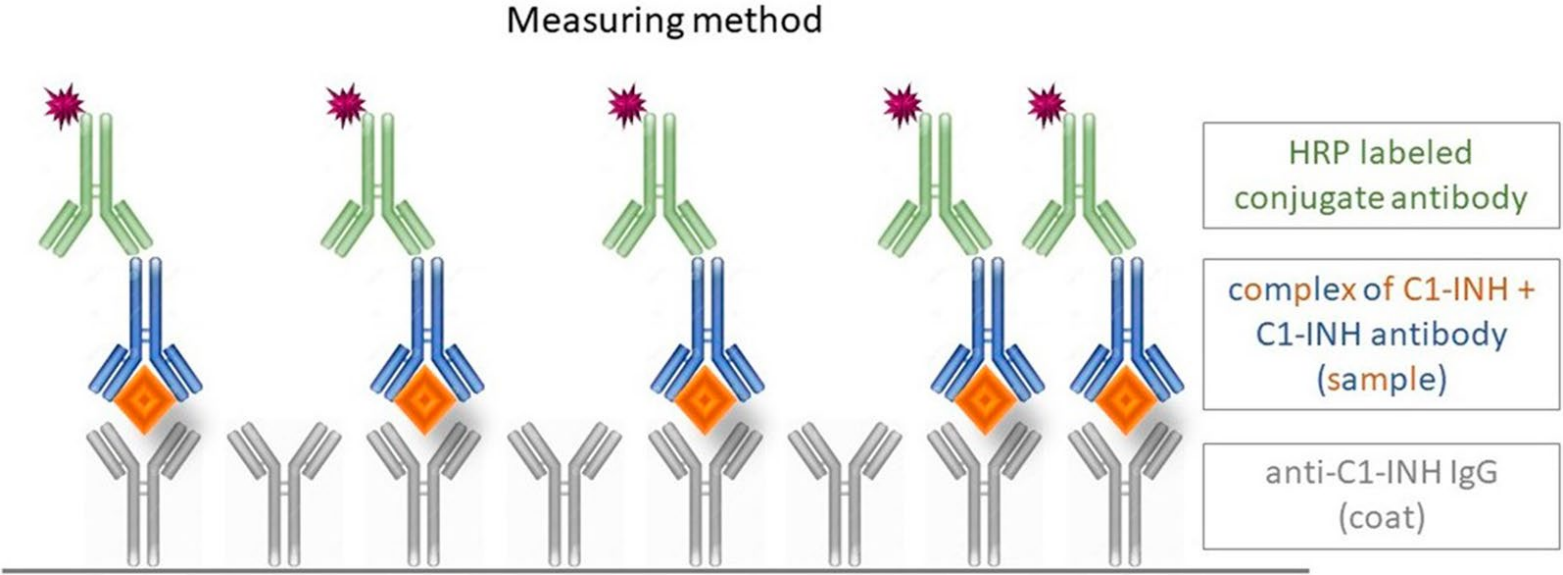
Measuring method for the C1-INH/C1-INH antibody complexes

The method is summarized on Fig. 2.

## Data Availability

The datasets used and/or analysed during the current study are available from the corresponding author on reasonable request.

## Abbreviations

C1-INH: C1-inhibitor
C1-INH-AAE: Acquired angioedema due to C1-inhibitor deficiency
C1-INH-Ab: Antibodies against C1-inhibitor
C1-INH-HAE: Hereditary angioedema due to C1-inhibitor deficiency
CAC: C1-inhibitor / C1-inhibitor antibody complexes
CH50: Total hemolytical complement
ELISA: Enzyme-linked immuno-sorbent assay
MGUS: Monocloncal gammopathy of undetermined significance
